# Assessing Perfluorooctane Sulfonate (PFOS) Toxicity and Carcinogenicity Through Zebrafish (*Danio rerio*) Xenograft Assays

**DOI:** 10.3390/toxics13121077

**Published:** 2025-12-14

**Authors:** Tessa Block, Joan Renee DeMaio, Lela Skopec, Margaret Ayers, Eric Glasgow

**Affiliations:** Department of Oncology, Georgetown University, 4000 Reservoir Road, Washington, DC 20057, USA; jrd154@georgetown.edu (J.R.D.); lks66@georgetown.edu (L.S.); mla128@georgetown.edu (M.A.)

**Keywords:** PFOS, PFAS, xenograft, environmental, contaminants, pollutants, carcinogenic, zebrafish, NAM, ACHN, Caki-1, LC_50_, MTC

## Abstract

Persistent environmental pollutants such as per- and poly-fluoroalkyl substances (PFAS) have been associated with a wide range of toxic effects, including cancer. There are over 12,000 PFAS compounds, which may act as carcinogens individually or in combinations. Therefore, efficient in vivo new approach models of carcinogenicity are needed for evaluating environmental contaminant compounds and chemical mixtures. Here, we use the larval zebrafish xenograft assay to identify tumor growth activity of perfluorooctane sulfonate (PFOS), a known carcinogenic PFAS. Dose–response curves for PFOS exposure were used to identify the Maximum Tolerated Concentration (MTC) and Lethal Concentration causing 50% death (LC_50_) under xenograft conditions. Zebrafish xenografts were established by injecting fluorescently labeled kidney cancer cells into the embryonic body cavity near the developing kidney, followed by treatment with PFOS at a concentration of 5%, 10%, and 20% of the MTC. When treated with PFOS, zebrafish xenografts using renal cell carcinoma (ACHN) cells and clear renal cell carcinoma (Caki-1) cells show dose-dependent changes in tumor area. This study is the first to directly show cancer-promoting activity of a PFAS, using a rapid in vivo zebrafish xenograft assay, and demonstrates the utility of this model for validation of predicted cancer-promoting properties of environmental contaminants.

## 1. Introduction

Per- and poly-fluoroalkyl substances (PFAS) are a diverse group of chemical compounds known as “forever chemicals.” These chemicals are used in microchips, jet engines, cars, batteries, medical devices, refrigeration systems, and many more everyday items, because their chemical makeup makes them ideal compounds for protection against heat, corrosion, water damage, and more. However, the prominence of carbon-fluorine bonds in PFAS imparts a bond strength that is not broken apart by natural processes, allowing them to exist indefinitely in our environment [1,2,3].

Current environmental protection agency efforts note that over 12,000 different PFAS compounds exist in the environment, indicating that they have extensively permeated our daily lives [4]. With a multitude of different structures and properties, PFAS compounds likely act in combination with one another, as well as with other environmental contaminants. Multiple studies have begun to explore chemical combinations through various toxicology assays and mixture-specific gene expression in zebrafish [5,6,7,8], but there remains a large gap in understanding the effects of these combinatorial chemical exposures. PFAS chemicals are heavily suspected to be carcinogens, so their ubiquitous presence in our environment leads to concerning questions about their impact on our health [9,10]. The buildup of PFAS in our environment has alarmingly caused every person today to have registered PFAS levels in their blood, suggesting signs of significant bioaccumulation [9,10,11]. From an extensive review of the published literature, Steenland and Winquist found that there is an association between PFAS and cancer, particularly testicular and kidney cancers [10].

One concerning PFAS compound is perfluorooctane sulfonate (PFOS). With an estimated half-life of 3.4 years [12], the average global levels of PFOS in blood samples are 20–30 ng/mL [13]. Compelling evidence suggests that PFOS affects the urogenital system and promotes kidney cancer. Epigenetic, transcription factor, and kidney biomarker signatures, serum analyses, and epidemiologic studies link PFOS exposure to chronic kidney disease and cancer [14,15,16].

Here, we aim to develop a complementary approach to screen chemicals for potential carcinogenic properties, utilizing zebrafish xenograft assays. Zebrafish xenograft models are extensively utilized for investigating cancer biology, developing and validating anti-cancer drugs, and producing clinical diagnostics for guiding personalized cancer therapeutics [17,18].

Xenograft methods involve injecting human cells into a targeted region of the developing zebrafish embryo, placing the embryo in the compound of interest, and observing the tumor throughout the duration of chemical exposure. Compared to other vertebrate models, zebrafish are an ideal model for translatable biomedical research because they have a fully sequenced genome, share over 70% of their genome with humans, and consist of transparent embryos allowing for easy developmental observation [19]. Our xenograft assay merits designation as a New Approach Methodology (NAM) as it is an animal xenograft that satisfies the desirable traits of human 3D culture while also allowing for systems analyses only possible in an animal model [20]. The proposed method is relatively high-throughput and could be used to efficiently screen thousands of chemicals or chemical mixtures for carcinogenic potential.

Our approach, using PFOS as a test compound, was to systematically evaluate toxicity at the exposure times and temperature expected to be used for the xenograft assays. From these experiments, we determined the Lethal Concentration 50 (LC_50_), defined as the median concentration resulting in 50% mortality, and the Maximum Tolerated Concentration (MTC), defined as the highest concentration that reliably results in 100% survival [21,22]. Zebrafish xenografts were then exposed to sublethal PFOS concentrations based on the garnered metrics.

Because PFOS exposure is linked to chronic kidney disease and cancer, we tested two well-studied kidney cancer cell lines. A renal cell carcinoma line, ACHN, was chosen as it is known to proliferate in zebrafish xenografts. A clear cell renal cell carcinoma line, Caki-1, was chosen as a model for the most common form of kidney cancer [23]. Fluorescently labeled versions of these cells were used for the xenografts and imaged over several time points to track changes in tumor size. When treated with PFOS, these kidney cancer cells show dose-dependent increases in tumor area compared to controls.

This study is the first to directly show cancer-promoting activity of PFOS, using a rapid in vivo zebrafish xenograft assay. Considering the popularity of zebrafish as toxicology models and the prevalence of zebrafish xenograft assays for cancer studies, it is somewhat surprising that these models are not commonly utilized for carcinogenesis screening of chemical compounds. Our results demonstrate the utility of zebrafish xenograft assays for the validation of predicted cancer-promoting properties of environmental contaminants, such as PFAS.

## 2. Materials and Methods

### 2.1. Zebrafish

Zebrafish husbandry, breeding, and injections were performed by the Georgetown-Lombardi Animal Shared Resource at Georgetown University, Washington, D.C. Zebrafish studies were conducted in accordance with NIH guidelines for the care and use of laboratory animals and were approved by the Georgetown University Institutional Animal Care and Use Committee. Protocol # 2017-0078.

The zebrafish facility is a custom-built Aquarius System from Aquatic Enterprises (Bridgewater, MA, USA). It is a recirculation system with a 10% water change per day. The light cycle is 14 h of light and 10 h of dark. Salinity and pH are kept between 450 and 500 microsiemens and 7.6–8.2, respectively, with automated dosing. Fish are fed twice daily a custom diet consisting of 57% tropical flake fish food, 10% krill powder, and 33% dechorionated brine shrimp cysts. Fish density is kept between 3 and 6 fish per liter.

Eggs were obtained with natural pairwise or group crosses in breeding chambers. The embryos were collected in the morning and transferred to fish water (0.3 g/L sea salt, Instant Ocean, Blacksburg, VA, USA) containing 200 μM phenylthiourea (PTU, Sigma-Aldrich, St. Louis, MO, USA) to inhibit pigment formation. The age of the embryos was determined using morphological staging criteria [24]. At 1 day post-fertilization (dpf), embryos were chemically dechorionated using 200 μg/mL pronase (CalBioChem, Sigma-Aldrich). A total of 700 fertilized embryos were used for the following assays.

### 2.2. Toxicology Assays

Perfluorooctane sulfonate (PFOS) was purchased from Sigma-Aldrich (CAS# 1763-23-1). A total of 200 mM PFOS stock solution in DMSO was made fresh on the day of the experiment and diluted to 2× final concentration in fish water containing PTU.

Dechorionated zebrafish embryos were arrayed into 96-well plates in 100 μL fish water containing PTU at 1 dpf. At 2 dpf, 100 μL of 2× PFOS solution was added, and plates were incubated at 33 °C for 24 h. In our study, 33 °C was chosen for xenograft experiments as a standard compromise temperature for mammalian cell growth and larva viability.

Embryo survival was assessed at 24 h post-treatment, corresponding to 72 h post-fertilization. If the embryo had physically deteriorated and/or there was no heartbeat, the embryo was assigned a 0 value to denote death. If the embryo showed any movement and/or a discernible heartbeat, the embryo was assigned a 1 value to denote survival. Range-finding experiments used 4 embryos per group for two repeats and 8 embryos per group for one repeat, performed on different days and with different breeding groups. Eight concentrations from 0.32 to 1000 μM were tested, as well as the inclusion of a PTU negative control and DMSO vehicle control group. The total *n* for the range-finding experiments was 160 embryos. Refined concentration experiments used 8 embryos per group, with 3 independent repeats. Eleven concentrations from 20–355 μM were tested, as well as the inclusion of a DMSO vehicle control group. The total *n* for the refined range experiments was 288 embryos. Analysis was performed using GraphPad Prism Software (version 10.6.0). The number of embryos scoring as 0 s and 1 s were averaged for each experimental group. A “Sigmoidal, 4PL, X is concentration” plot was generated using the nonlinear fit analysis function, and graphical visualizations were created. From this fit, LC_50_s were calculated. MTCs were calculated from the nonlinear fit by taking 75% of the concentration where 90% percent survival would be reached.

Our MTC determination aimed to quantifiably identify the point on the nonlinear fit where the curve begins to deviate from 100 percent, a metric that is similar to a typical MTC, or No Observed Adverse Effect Level (NOAEL), determination. Our quantifiable method for MTC determination enabled the rapid determination of likely carcinogenic concentrations of test compounds.

Once the MTC was established, derived test concentrations of 5%, 10%, and 20% of the MTC, as well as a positive control of 265 μM and a vehicle control of 0.002% DMSO, were tested on 8 embryos per group to assess whether the larvae could survive up to three days of exposure at these levels, mimicking the length of our designed xenograft. Two biological replicates of each test group were performed. A total of 80 embryos were used for deriving our xenograft test concentrations.

### 2.3. Cell Culture

ACHN (CRL-1611) cell cultures were obtained from the Georgetown University Tissue Culture Shared Resource. ACHN cells were labeled with 1 μM Vibrant DiI cell-labeling solution (Invitrogen, Eugene, OR, USA, V22885) at 1 × 10^6^ cells/mL, incubated for 5 min at 37 °C, and then placed on ice for 15 min. Labeled cells were washed twice with PBS, once in 0.5 mM EDTA in PBS, and then resuspended in 10 μL 0.5 mM EDTA in PBS for loading the injection needle. Stably GFP-expressing Caki-1 (HTB-46) cells were purchased from Innoprot and cultured according to the manufacturer’s protocol.

### 2.4. Zebrafish Xenografts

Zebrafish transgenic *Tg(kdrl:grcfp)zn1* or *Tg(mpeg1:mCherry)gl23* embryos at 2 dpf were anesthetized with 0.016% tricaine (Sigma-Aldrich) in fish water, loaded on to an agarose injection plate, and injected with 100–200 cells into the body above the yolk sack just posterior to the pectoral fin, using an air driven Picospritzer II microinjector (General Valve Corp, Fairfield, NJ, USA) under a Leica M165 stereoscope. Following injection, embryos were examined for proper injection size, location, and absence of cells in the tail. Correctly injected embryos were imaged on a Leica M165 stereomicroscope with a K5 CMOS camera, and arrayed into 96-well plates with one embryo per well in 100 μL PTU fish water. A total of 100 μL of 2× PFOS solution at an appropriate concentration was added to the well for a final volume of 200 μL per embryo. Each concentration and a negative control of PTU included 2–4 trials of *n* = 8. The embryos were incubated at 33 °C for three days and then re-imaged. The included *n* values in the xenograft results represent the embryos that survived injection and incubation. Embryos that did not survive to the three-day mark were excluded from the final analysis. The area of labeled cells was measured from the day 0 and day 3 images of each embryo using ImageJ (version 1.54p) [25]. Graphs were made using GraphPad Prism Software (version 10.6.0).

### 2.5. Statistical Analysis

A one-way analysis of variance (ANOVA) test with multiple comparisons of means was selected as the primary statistical analysis for the xenograft experiments. All statistical analyses were performed with a confidence level of 95% (α = 0.05) assuming a normal Gaussian distribution. Multiple comparisons were performed using the uncorrected Fisher’s LSD test to compare the means of all treatment groups pairwise. Assumption testing was completed. The ACHN ANOVA validated the assumption of homogeneity of variances via the Brown–Forsythe test (F(DFN, DFd) = 1.735 (3, 57), *p* = 0.1700) and Bartlett’s test (F = 3.512, *p* = 0.3192). It also validated the normality of residuals assumption via the D’Agostino–Pearson omnibus (K2) test (K2 = 4.041, *p* = 0.1326). The Caki-1 ANOVA met the normality of residuals assumption via the same test (K2 = 5.734, *p* = 0.0569) but did not show a significant homogeneity of variances via the Brown–Forsythe or Bartlett’s test. For this reason, a Brown–Forsythe ANOVA with multiple uncorrected, unpaired Welch’s *t*-tests was performed for the Caki-1 set of data. All statistical analysis was performed using GraphPad Prism Software (version 10.6.0).

All statistical values from these tests can be found in Supplemental Tables S2–S5. Adjustments were not applied for multiple comparisons, as our study of a new methodology planned for only six set comparisons of pre-specified concentrations. We chose to evaluate all pairwise comparisons to gain insight into dose-dependency, but please refer to Supplemental Table S5 for post hoc Dunnett’s test analyses of the treatment groups compared to the control group for each cell line.

## 3. Results

### 3.1. Toxicology Experiments

To determine the optimal dose for treating zebrafish xenografts with PFOS, we investigated dose–response toxicology under conditions that should be used for the xenograft assays. We initially performed a range-finding assay to determine the interval where the compound became lethal to the embryos. We then used that interval to inform our refined range experiment. We chose one day of treatment to mitigate variability in our results.

**Figure 1:**
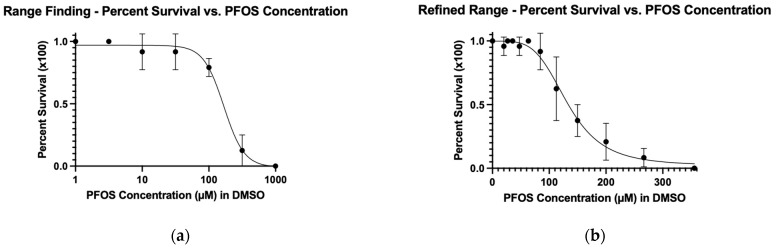
The (**a**) range-finding (R^2^ = 96.15) and (**b**) refined-range (R^2^ = 94.48) dose–response curves used to obtain LC_50_ and MTC data for PFOS. Embryos were treated at 2 dpf for 24 h at 33 °C and then assessed.

To determine the LC_50_, we used a nonlinear fit for both the range-finding and refined range experiments (Figure 1). The LC_50_ from the range-finding assay was 167 μM, and the refined LC_50_ was 132 μM (Table 1). The LC_50_ values were calculated for the range-finding and refined experiments to evaluate how closely the values from these two experiments matched. While not wildly different, in our view, these values are different enough to warrant performing a refined range experiment in order to calculate appropriate concentrations for xenograft assays.

Despite our conditions, 24 h of treatment at 2 dpf and incubation at 33 °C, being different from those reported in the literature, our LC_50_ of 132 μM is within the range of consensus values (Supplementary Table S1). This was somewhat surprising given that most assays begin treatment within hours of fertilization.

**Figure 2:**
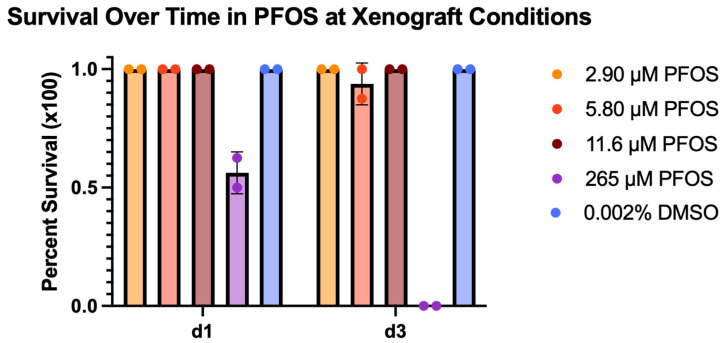
Survival under xenograft conditions from exposure to expected tolerated concentrations of PFOS. A total of 0.002% DMSO was used as a vehicle control, and 265 µM PFOS was used as a positive control. Assessment of survival was performed on 2 dpf embryos raised at 33 °C after 1 day of treatment and 3 days of treatment. Each test group included two biological repeats with *n* = 8 for each.

Once we determined the PFOS LC_50_ value, we established the corresponding MTC. We defined the MTC as 75% of the concentration that resulted in 90% survival. These calculations resulted in an MTC of 58 μM PFOS for the refined range experiment. Treatment concentrations based on 5%, 10%, and 20% of the PFOS MTC were chosen for the xenograft experiments (Table 1). These concentrations were experimentally tested under xenograft conditions to confirm embryo survival of greater than 90% for 3 days of treatment (Figure 2).

### 3.2. Xenograft Experiments

After confirming that the 2.9, 5.8, and 11.6 µM PFOS treatment groups all had survival rates greater than 90%, these concentrations were chosen for the xenograft assays. ACHN cells were labelled with DiI and injected into the area of the developing kidney. The tumors were imaged on the day of injection (d0) and treated with PFOS. They were then re-imaged on day 3 post-injection.

**Figure 3:**
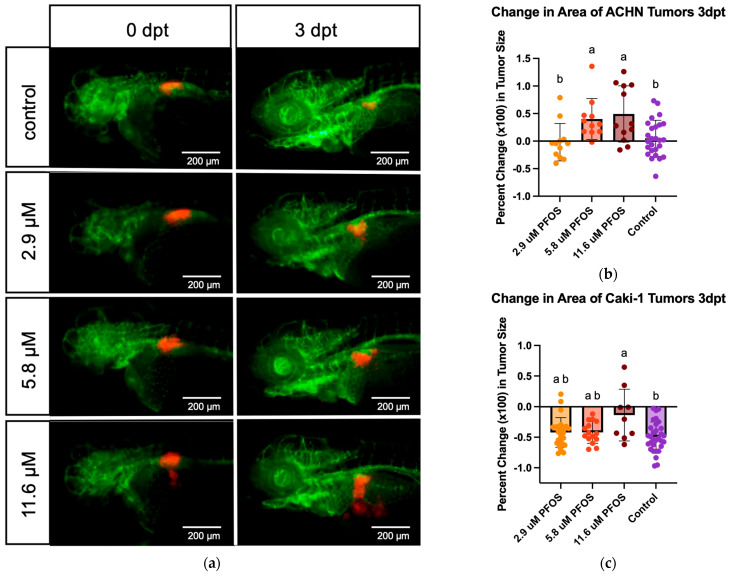
(**a**) Red fluorescent kidney cancer ACHN cell xenografts overlaid on *Tg(kdrl:GCRFP)* transgenic embryos with green blood vessels. DiI-labeled cells were injected dorsally to the yolk in the area of the developing kidney of 2 dpf embryos. Images at 0 and 3 days post PFOS treatment (dpt). (**b**) The percent change in area of ACHN tumors of PFOS-treated xenograft groups after 3 days of chemical treatment. Statistical significance was tested via a one-way ANOVA (α = 0.05) with Fisher’s LSD multiple comparisons test, all pairwise. Different letters above the bars represent significant group differences (*p* ≤ 0.05) (starting *n* values—2.9 µM: 16, 5.8 µM: 20, 11.6 µM: 16, Control: 32; final *n* values—2.9 µM: 12, 5.8 µM: 11, 11.6 µM: 12, Control: 26). (**c**) The percent change in area of Caki-1-GFP tumors of PFOS-treated xenograft groups after 3 days of chemical treatment. Statistical significance was tested via a Brown–Forsythe ANOVA (α = 0.05) with multiple unpaired Welch’s *t*-tests, all pairwise. Different letters above the bars represent significant group differences (*p* ≤ 0.05). (starting *n* values—2.9 µM: 24, 5.8 µM: 16, 11.6 µM: 16, Control: 32; final *n* values—2.9 µM: 24, 5.8 µM: 15, 11.6 µM: 9, Control: 31).

Representative images of ACHN cell xenografts at 0 and 3 days post-treatment/injection are shown in Figure 3a. Tumor areas measured using ImageJ were used to calculate the percent change in tumor size. The percent change for the embryos of each treatment group was averaged and plotted as percent change in tumor size. The figure shows a dose-dependent increase in tumor growth in ACHN cells.

In support of the visual in Figure 3a, an analysis of variance for the ACHN groups revealed a statistically significant difference in the groups’ mean percent increase in tumor areas (F(DFn, DFd) = F(3, 57) = 6.107, *p* = 0.0011). Subsequent Fisher’s LSD tests found that the 11.6 µM treatment group mean percent increase in tumor area was significantly higher than in the control group (M = 0.4928, SD = 0.5097 vs. M = 0.05064, SD = 0.3271, t(57) = 3.347, *p* = 0.0015). The 5.8 µM treatment group’s mean percent increase in tumor area was also significantly higher than in the control group (M = 0.4015, SD = 0.3706 vs. M = 0.05064, SD = 0.3271, t(57) = 2.577, *p* = 0.0126). The mean percent increase in tumor area of the 11.6 µM group was also significantly higher than in the 2.9 µM group (M = 0.4928, SD = 0.5097 vs. M = −0.01985, SD = 0.3387, t(57) = 3.317, *p* = 0.0016). Finally, the mean percent increase in tumor area of the 5.8 µM group was significantly higher than in the 2.9 µM group (M = 0.4015, SD = 0.3706 vs. M = −0.01985, SD = 0.3387, t(57) = 2.666, *p* = 0.01). There was no statistically significant difference between the mean percent increase in tumor size between the control group to the 2.9 µM group (t(57) = 0.5336, *p* = 0.5957) and the 5.8 µM group to the 11.6 µM group (t(57) = 0.5784, *p* = 0.5653). Figure 3b depicts these comparisons using compact letter denotation [26,27].

To test a second kidney cancer cell line, Caki-1 cells expressing GFP were injected into the area of the developing kidney. The tumors were imaged on the day of injection (d0) and treated with PFOS, then imaged on day 3 post-injection (Supplemental Figure S1). Tumor areas were again measured using ImageJ and used to calculate the percent change in tumor size. The percent change for the embryos of each treatment group was averaged and plotted as percent change in tumor size, shown in Figure 3c.

Figure 3c reveals a dose-dependent protective effect for tumor growth in Caki-1 cells. The Caki-1 analysis of variance revealed a statistically significant difference in the groups’ mean percent change in tumor areas (F*(DFn, DFd) = 3.412 (3.000, 20.64), *p* = 0.0367). Subsequent unpaired Welch’s *t*-tests found that the 11.6 µM treatment group mean percent change in tumor area was statistically less negative than the control group (M = −0.1365, SD = 0.4236 vs. M = −0.4942, SD = 0.2442, t(9.594) = 2.419, *p* = 0.0371). The control group showed statistically similar percent change in tumor area compared to the 2.9 µM (M = −0.4942, SD = 0.2442 vs. M = −0.4218, SD = 0.2437, t(49.65) = 1.092, *p* = 0.2799) and the 5.8 µM (M = −0.4942, SD = 0.2442 vs. M = −0.4209, SD = 0.1769, t(37.03) = 1.158, *p* = 0.2543) groups. The 11.6 µM group showed statistically similar percent change in tumor area to the 5.8 µM group (t(9.701) = 1.916, *p* = 0.0852) and the 2.9 µM group (t(10.06) = 1.905, *p* = 0.0857). The 5.8 µM group also saw statistically similar percent change in tumor area to the 2.9 µM group (t(36.05) = 0.01298, *p* = 0.9897). Figure 3c depicts these comparisons using letter denotation.

## 4. Discussion

Links between PFOS exposure and kidney cancer have been studied utilizing a variety of techniques including proteomics, cell culture, organoid studies, and more. None of these studies, however, directly tested the cancer-promoting properties of PFOS in vivo. Our goal was to establish an efficient protocol for testing environmental contaminants for cancer-promoting activity using zebrafish xenograft assays.

For the development of our xenograft procedure, we determined appropriate PFOS concentrations for our assay conditions. We calculated an LC_50_ of 167 μM from the range-finding assay and a refined LC_50_ of 132 μM after 24 h of treatment at 33 °C, starting at 2 dpf. Our refined value is a 0.8 factor of our range-finding value. LC_50_ values for similar toxicity assays reported in the literature lie in a consensus range from 102 to 136 μM at 72 h post-fertilization (hpf) (see Supplemental Table S1). These experiments generally start treatment soon after fertilization, use PFOS potassium salt or PFOS as a compound, and variably make stocks in DMSO or water. Inconsistent LC_50_s may also be due to an overly wide range of test concentrations, differences in solvents, a low number of test subjects, contrasting assessment timepoints, incubation temperature, and distinct compound formulations. Overall, though, compared to previous studies, the refined LC_50_ value obtained in this study falls within the expected range.

We chose to measure toxicology based on embryo survival because this is the fastest and most unambiguous method. Several studies have measured PFOS toxicity in zebrafish utilizing more sensitive assays such as behavioral analyses [28,29], gene expression signatures [30,31,32], and altered developmental morphology [33,34,35] (Supplemental Table S1). Our toxicology experiments were assessed at 24 h of treatment versus the 3-day mark used for our xenografts. The reasoning for this decision was based on our objective to establish an efficient process for evaluating environmental contaminants. The 24 h treatment assessment period reduces variability and is faster despite requiring that the test concentrations be experimentally confirmed for the xenograft assays (see Figure 2).

Background PFAS contamination from labware and a laboratory fish diet is a concern, particularly for evaluating LC_50_ and EC_50_ measurements among different laboratories [36,37]. However, these factors are unlikely to influence the results of the xenograft studies since those experiments utilize experimental and control groups exposed to identical PFAS background levels.

While zebrafish are commonly utilized for toxicology studies, zebrafish xenograft assays to screen for carcinogenic activity of environmental toxins have not been utilized. These assays are commonly used for evaluating cancer cell biology, chemosensitivity, radiosensitivity, angiogenesis, and immune cell–tumor interactions [17,18]. Zebrafish xenografts implanted with patient biopsies are being developed to test patient tumor-specific chemosensitivity and immunotherapies. Importantly, several recent papers have demonstrated high predictive value of these xenograft assays for patient response to therapy [38,39,40,41,42,43]. Our results are in line with the biologically meaningful endpoints seen in well-established cancer-therapeutic xenograft studies. Given the demonstrated high predictive value of zebrafish xenografts for human tumor progression, our objective was to test whether this assay could also identify potential carcinogenic environmental contaminants, using PFOS as a test case compound.

We focused our effort on kidney cancer because there is mounting evidence of an association of PFAS with cancer, particularly with testicular and kidney cancer [10]. Additionally, the National Institutes of Health (NIH) Division of Cancer Epidemiology and Genetics (DCEG) has used banked serum specimens to connect PFAS presence to kidney cancer development [15]. Wen et al. saw, in collected mouse kidney samples, increased kidney injury markers Acta2 and Bcl2l1, upregulated transcription factors-Nef2l2, Hes1, Ppara, and Ppard-downregulated transcription factors-Smarca2 and Pparg-decreased global DNA methylation, and histone demethylase gene upregulation. In addition, they saw dose-dependent accumulation of PFOS in the mouse kidney [14].

For our xenograft experiments, we utilized two kidney cancer cell lines, ACHN and Caki-1 cells. Xenografts with ACHN cells showed significant tumor growth when treated with PFOS. The 11.6 μM and 5.8 μM treatment groups showed a statistically significant percent increase in tumor area compared to the control group while the lowest test concentration did not. The image analysis results support a PFOS concentration-dependent increase in tumor area. The observed dose-dependency is supported by increasing statistical confidence with increasing concentration (*p*-value comparison of the control to 11.6 μM < 5.8 μM < 2.9 μM).

With Caki-1 cells, the xenograft assay showed a PFOS-dependent protection from the decreased tumor size that was observed in the control group. As seen in the ACHN experiment, the 11.6 μM treatment group showed a statistically different percent change in tumor area compared to the control. However, the two lower test concentrations did not. Again, from visuals and a correlation between statistical confidence and PFOS concentration, our confidence that PFOS carcinogenic activity acts in a dose-dependent manner has increased.

ACHN cells were selected for these studies because they had been shown to proliferate in zebrafish xenograft assays, which we confirm, as well as demonstrate growth-promoting properties of PFOS in these cells [23]. Caki-1 cells, on the other hand, have not previously been utilized for zebrafish xenografts, but are considered a model for the most common form of kidney cancer, clear cell carcinoma. In zebrafish xenografts, Caki-1 cells do not proliferate well, resulting in a net shrinkage of the xenograft tumor, as shown here. However, even though the tumor is shrinking, Caki-1 cells respond predictably to treatment with chemotherapeutic agents (unpublished observations, E.G.). Remarkably, our results show a protective effect of PFOS on Caki-1 tumor shrinkage. Tumor growth or shrinkage in zebrafish xenografts is due to the sum of factors, including proliferation rates, apoptotic cell death rates, innate immune-directed tumor cell death, or protection [39,44,45]. While our experiments do not distinguish between these mechanisms, the positive effect of PFOS on tumor growth in ACNH cells and the protective effects of this compound on Caki-1 tumor shrinkage demonstrate carcinogenic effects during net growth or shrinkage of kidney cancer cells in vivo.

Visual inspection and image analysis of our xenograft results support the idea that while PFOS typically presents as toxic to cells, some characteristic of the compound is stimulating or protecting tumor growth. While our xenograft protocol uses short exposure times, which are unlikely to reflect typical exposure conditions in humans, it does demonstrate that PFOS exposure can promote cancerous cell behavior. On average, about 20–30 ng/mL or 0.040–0.060 μM PFOS is detected in the human bloodstream [13]. While only a twentieth of the maximum concentration used for our xenograft assay, the connection between the cancer-promoting abilities of PFOS and levels of PFOS identified in human blood is concerning. Bioaccumulation in the kidney could result in concentrations greater than those measured in blood. It should also be noted that our study focused solely on short-term exposure. The results obtained in this study cannot be directly compared to chronic studies. However, our observation of the cancer-promoting properties of PFOS under these conditions propound concern with chronic exposures. Importantly, while the present studies do not investigate specific mechanisms for PFOS-dependent cancer growth, our xenograft protocol provides a platform in which mechanistic investigation can be efficiently pursued.

The conclusions from our xenograft studies are supported by several in vitro studies. Cell proliferation, flow cytometry, immunocytochemistry, cell migration, and invasion assays demonstrate the potential tumorigenic activity of PFOS [46]. In three-dimensional colorectal cancer spheroids treated with PFOS, molecular analysis showed downregulation of E-cadherin and upregulation of N-cadherin and vimentin [47]. In contrast to spheroid cultures, a powerful aspect of our in vivo zebrafish xenograft assay is that it encompasses the entire animal environment, integrating effects on cancer cells, the cancer microenvironment, innate immune cells, compound distribution, and metabolism. Importantly, PFAS molecules induce comparable transcriptional changes and affect the same metabolic processes across inter-species borders [48]. In mice, PFOS treatment resulted in an accumulation of the compound in the kidney, increased kidney injury markers, and global DNA hypomethylation, all of which support a potentially cancerous effect of the compound [14].

## 5. Conclusions

Overall, this study provides a new in vivo model to efficiently identify potential carcinogenic properties of environmental contaminants, and specifically supports the proposal that PFOS promotes urogenital cancer. These results bolster the potential classification of PFOS as a carcinogen. With a pressing need to study the carcinogenic potential of environmental contaminants, zebrafish xenografts emerge as an extremely effective tool for analysis. This study focused on tumor growth of two cell lines resulting from exposure to one PFAS compound. Future research can expand upon these findings by replicating them with cell lines from different cancer types. Our zebrafish xenograft protocol is especially applicable for screening chemical combinations. There exist over 12 thousand toxic PFAS compounds whose mixtures, and their subsequent unknown effects, are endless. Computational chemists have already begun developing models to identify such mixtures of concern [49,50]. Our xenograft protocol is ideally suited as an efficient method to validate computationally predicted mixtures and identify carcinogenic potential of environmental contaminants.

## Figures and Tables

**Table 1:** PFOS LC_50_ and MTC data and calculated concentrations for xenograft assays.

Sample Set	LC_50_ (μM)	MTC (μM)	5% of MTC (μM)	10% of MTC (μM)	20% of MTC (μM)
Range-Finding	167	73	-	-	-
Refined Range	132	58	2.9	5.8	11.6

## Data Availability

The original contributions presented in this study are included in the article/Supplementary Materials. Further inquiries can be directed to the corresponding authors.

## Supplementary Materials

The following supporting information can be downloaded at https://www.mdpi.com/article/10.3390/toxics13121077/s1; Supplemental Figure S1 includes representative images of Caki-1 xenografts. Supplemental Table S1 summarizes the literature concerning PFOS and zebrafish. Supplemental Tables S2–S5 include all xenograft statistical analysis outputs.

## Abbreviations

The following abbreviations are used in this manuscript:

PFOS	Perfluorooctane sulfonate
PFAS	Per- and Poly-fluoroalkyl substances
LC_50_	Lethal Concentration causing 50% death
MTC	Maximum Tolerated Concentration
ACHN	Renal cell carcinoma
Caki-1	Clear renal cell carcinoma cells
NAM	New Approach Methodology
hpf	Hours post-fertilization
dpf	Days post-fertilization
dpt	Days post-treatment
PTU	Phenylthiourea
ANOVA	One-way analysis of variance
NOAEL	No Observed Adverse Effect Level
NIH	National Institutes of Health
DCEG	Division of Cancer Epidemiology and Genetics
