# Supplementary material for: Assessing Perfluorooctane Sulfonate (PFOS) Toxicity and Carcinogenicity Through Zebrafish (Danio rerio) Xenograft Assays
Source: Toxics. 2025 Dec 14;13(12):1077. doi: 10.3390/toxics13121077 (PMC12737301; doi:10.3390/toxics13121077)
Supplement: Supplementary file 1 [file toxics-13-01077-s001.zip › File S1_Raw Data.pdf]

Range Finding Toxicology Curve

Plate Layout:

(Trial 1)	1	2	3	4	5	6	7	8	9	10	11	12
A	1000	316.22777	100	31.622777	10	3.1622777	1	0.3162278			Control	Control + 0.5% DMSO
B	1000	316.22777	100	31.622777	10	3.1622777	1	0.3162278			Control	Control + 0.5% DMSO
C	1000	316.22777	100	31.622777	10	3.1622777	1	0.3162278			Control	Control + 0.5% DMSO
D	1000	316.22777	100	31.622777	10	3.1622777	1	0.3162278			Control	Control + 0.5% DMSO
E (Trial 2)	1000	316.22777	100	31.622777	10	3.1622777	1	0.3162278			Control	Control + 0.5% DMSO
F	1000	316.22777	100	31.622777	10	3.1622777	1	0.3162278			Control	Control + 0.5% DMSO
G	1000	316.22777	100	31.622777	10	3.1622777	1	0.3162278			Control	Control + 0.5% DMSO
H	1000	316.22777	100	31.622777	10	3.1622777	1	0.3162278			Control	Control + 0.5% DMSO

(Trial 3)	1	2	3	4	5	6	7	8	9	10	11	12
A	1000	316.22777	100	31.622777	10	3.1622777	1	0.3162278			Control	Control + 0.5% DMSO
B	1000	316.22777	100	31.622777	10	3.1622777	1	0.3162278			Control	Control + 0.5% DMSO
C	1000	316.22777	100	31.622777	10	3.1622777	1	0.3162278			Control	Control + 0.5% DMSO
D	1000	316.22777	100	31.622777	10	3.1622777	1	0.3162278			Control	Control + 0.5% DMSO
E	1000	316.22777	100	31.622777	10	3.1622777	1	0.3162278			Control	Control + 0.5% DMSO
F	1000	316.22777	100	31.622777	10	3.1622777	1	0.3162278			Control	Control + 0.5% DMSO
G	1000	316.22777	100	31.622777	10	3.1622777	1	0.3162278			Control	Control + 0.5% DMSO
H	1000	316.22777	100	31.622777	10	3.1622777	1	0.3162278			Control	Control + 0.5% DMSO

Results:

(Trial 1)	1	2	3	4	5	6	7	8	9	10	11	12
A	0	0	0	1	1	1	1	1			1	1
B	0	0	1	1	1	1	1	1			1	1
C	0	0	1	1	1	1	1	1			1	1
D	0	0	1	1	0	1	1	1			1	1
E (Trial 2)	0	0	0	1	1	1	1	1			1	1
F	0	0	1	1	1	1	1	1			1	1
G	0	0	1	1	1	1	1	1			1	1
H	0	1	1	1	1	1	1	1			1	1

(Trial 3)	1	2	3	4	5	6	7	8	9	10	11	12
A	0	0	1	1	1	1	1	1			1	1
B	0	1	0	0	1	1	1	1			1	1
C	0	0	1	1	1	1	1	1			1	1
D	0	0	1	0	1	1	1	1			1	1
E	0	0	1	1	1	1	1	1			1	1
F	0	0	1	1	1	1	1	1			1	1
G	0	0	1	1	1	1	1	1			1	1
H	0	0	1	1	1	1	1	1			1	1

Analysis:

PFOS (uM)	1000	316.227766	100	31.6227766	10	3.16227766	1	0.31622777	Control + 0.5% DMSO
Replicate 1	0	0	0.75	1	0.75	1	1	1	1
Replicate 2	0	0.25	0.75	1	1	1	1	1	1
Replicate 3	0	0.125	0.875	0.75	1	1	1	1	1
Average	0	0.125	0.7916666667	0.9166666667	0.9166666667	1	1	1	1
Std. Dev	0	0.125	0.072168783[illegible]	0.144337567[illegible]	0.144337567[illegible]	0	0	0	0

Refined Range Toxicology Curve

Plate Layout:

(Trial 1)	1	2	3	4	5	6	7	8	9	10	11	12
A	Control + 0.18% DMSO	20	26.67042864	35.5655882	47.42747411	63.2455532	84.33930069	112.468265	149.9788419	200	266.7	355.66
B	Control + 0.18% DMSO	20	26.67042864	35.5655882	47.42747411	63.2455532	84.33930069	112.468265	149.9788419	200	266.7	355.66
C	Control + 0.18% DMSO	20	26.67042864	35.5655882	47.42747411	63.2455532	84.33930069	112.468265	149.9788419	200	266.7	355.66
D	Control + 0.18% DMSO	20	26.67042864	35.5655882	47.42747411	63.2455532	84.33930069	112.468265	149.9788419	200	266.7	355.66
E	Control + 0.18% DMSO	20	26.67042864	35.5655882	47.42747411	63.2455532	84.33930069	112.468265	149.9788419	200	266.7	355.66
F	Control + 0.18% DMSO	20	26.67042864	35.5655882	47.42747411	63.2455532	84.33930069	112.468265	149.9788419	200	266.7	355.66
G	Control + 0.18% DMSO	20	26.67042864	35.5655882	47.42747411	63.2455532	84.33930069	112.468265	149.9788419	200	266.7	355.66
H	Control + 0.18% DMSO	20	26.67042864	35.5655882	47.42747411	63.2455532	84.33930069	112.468265	149.9788419	200	266.7	355.66

(Trial 2)										10	11	12
A	Control + 0.18% DMSO	20	26.67042864	35.5655882	47.42747411	63.2455532	84.33930069	112.468265	149.9788419	200	266.7	355.66
B	Control + 0.18% DMSO	20	26.67042864	35.5655882	47.42747411	63.2455532	84.33930069	112.468265	149.9788419	200	266.7	355.66
C	Control + 0.18% DMSO	20	26.67042864	35.5655882	47.42747411	63.2455532	84.33930069	112.468265	149.9788419	200	266.7	355.66
D	Control + 0.18% DMSO	20	26.67042864	35.5655882	47.42747411	63.2455532	84.33930069	112.468265	149.9788419	200	266.7	355.66
E	Control + 0.18% DMSO	20	26.67042864	35.5655882	47.42747411	63.2455532	84.33930069	112.468265	149.9788419	200	266.7	355.66
F	Control + 0.18% DMSO	20	26.67042864	35.5655882	47.42747411	63.2455532	84.33930069	112.468265	149.9788419	200	266.7	355.66
G	Control + 0.18% DMSO	20	26.67042864	35.5655882	47.42747411	63.2455532	84.33930069	112.468265	149.9788419	200	266.7	355.66
H	Control + 0.18% DMSO	20	26.67042864	35.5655882	47.42747411	63.2455532	84.33930069	112.468265	149.9788419	200	266.7	355.66

(Trial 3)	1	2	3	4	5	6	7	8	9	10	11	12
A	Control + 0.18% DMSO	20	26.67042864	35.5655882	47.42747411	63.2455532	84.33930069	112.468265	149.9788419	200	266.7	355.66
B	Control + 0.18% DMSO	20	26.67042864	35.5655882	47.42747411	63.2455532	84.33930069	112.468265	149.9788419	200	266.7	355.66
C	Control + 0.18% DMSO	20	26.67042864	35.5655882	47.42747411	63.2455532	84.33930069	112.468265	149.9788419	200	266.7	355.66
D	Control + 0.18% DMSO	20	26.67042864	35.5655882	47.42747411	63.2455532	84.33930069	112.468265	149.9788419	200	266.7	355.66
E	Control + 0.18% DMSO	20	26.67042864	35.5655882	47.42747411	63.2455532	84.33930069	112.468265	149.9788419	200	266.7	355.66
F	Control + 0.18% DMSO	20	26.67042864	35.5655882	47.42747411	63.2455532	84.33930069	112.468265	149.9788419	200	266.7	355.66
G	Control + 0.18% DMSO	20	26.67042864	35.5655882	47.42747411	63.2455532	84.33930069	112.468265	149.9788419	200	266.7	355.66
H	Control + 0.18% DMSO	20	26.67042864	35.5655882	47.42747411	63.2455532	84.33930069	112.468265	149.9788419	200	266.7	355.66

Results:

(Trial 1)	1	2	3	4	5	6	7	8	9	10	11	12
A	1	1	1	1	1	1	1	1	1	1	1	0
B	1	1	1	1	1	1	1	1	1	0	0	0
C	1	1	1	1	1	1	1	1	0	0	0	0
D	1	0	1	1	1	1	1	1	0	0	0	0
E	1	1	1	1	1	1	1	0	0	0	0	0
F	1	1	1	1	1	1	1	1	0	0	0	0
G	1	1	1	1	1	1	1	1	1	0	0	0
H	1	1	1	1	1	1	1	1	0	0	0	0

(Trial 2)	1	2	3	4	5	6	7	8	9	10	11	12
A	1	1	1	1	1	1	1	1	1	0	0	0
B	1	1	1	1	1	1	1	1	1	0	0	0
C	1	1	1	1	1	1	1	1	0	0	0	0
D	1	1	1	1	1	1	1	0	0	0	0	0
E	1	1	1	1	1	1	1	0	0	0	0	0
F	1	1	1	1	1	1	1	0	0	0	0	0
G	1	1	1	1	1	1	1	1	1	0	0	0
H	1	1	1	1	0	1	1	1	1	1	0	0

(Trial 3)	1	2	3	4	5	6	7	8	9	10	11	12
A	1	1	1	1	1	1	1	0	1	1	1	0
B	1	1	1	1	1	1	1	1	1	0	0	0
C	1	1	1	1	1	1	1	0	0	0	0	0
D	1	1	1	1	1	1	0	0	0	1	0	0
E	1	1	1	1	1	1	1	0	0	0	0	0
F	1	1	1	1	1	1	0	1	0	0	0	0
G	1	1	1	1	1	1	1	1	0	0	0	0
H	1	1	1	1	1	1	1	0	0	1	0	0

Analysis:

PFOS (uM)	ontrol + 0.18% DMSO	20	26.67042864	35.5655882	47.42747411	63.2455532	84.33930069	112.468265	149.9788419	200	266.7	355.66
Replicate 1	1	0.875	1	1	1	1	1	0.875	0.375	0.125	0.125	0
Replicate 2	1	1	1	1	1	1	0.75	0.375	0.25	0.375	0.125	0
Replicate 3	1	1	1	1	0.875	1	1	0.625	0.5	0.125	0	0
Average	1	0.9583333333	1	1	0.9583333333	1	0.9166666667	0.625	0.375	0.2083333333	0.0833333333	0
Std. Dev	0	0.0721687836	0	0	0.0721687836	0	0.1443375673	0.25	0.125	0.1443375673	0.0721687836	0

**Plate Layout**											
	1	2	3	4	5	6	7	8	9	10	11	12
A	2.9 uM PFOS	5.8 uM PFOS	11.6 uM PFOS	265 uM PFOS	Cntrl	2.9 uM PFOS	5.8 uM PFOS	11.6 uM PFOS	265 uM PFOS	Cntrl		
B	2.9 uM PFOS	5.8 uM PFOS	11.6 uM PFOS	265 uM PFOS	Cntrl	2.9 uM PFOS	5.8 uM PFOS	11.6 uM PFOS	265 uM PFOS	Cntrl		
C	2.9 uM PFOS	5.8 uM PFOS	11.6 uM PFOS	265 uM PFOS	Cntrl	2.9 uM PFOS	5.8 uM PFOS	11.6 uM PFOS	265 uM PFOS	Cntrl		
D	2.9 uM PFOS	5.8 uM PFOS	11.6 uM PFOS	265 uM PFOS	Cntrl	2.9 uM PFOS	5.8 uM PFOS	11.6 uM PFOS	265 uM PFOS	Cntrl		
E	2.9 uM PFOS	5.8 uM PFOS	11.6 uM PFOS	265 uM PFOS	Cntrl	2.9 uM PFOS	5.8 uM PFOS	11.6 uM PFOS	265 uM PFOS	Cntrl		
F	2.9 uM PFOS	5.8 uM PFOS	11.6 uM PFOS	265 uM PFOS	Cntrl	2.9 uM PFOS	5.8 uM PFOS	11.6 uM PFOS	265 uM PFOS	Cntrl		
G	2.9 uM PFOS	5.8 uM PFOS	11.6 uM PFOS	265 uM PFOS	Cntrl	2.9 uM PFOS	5.8 uM PFOS	11.6 uM PFOS	265 uM PFOS	Cntrl		
H	2.9 uM PFOS	5.8 uM PFOS	11.6 uM PFOS	265 uM PFOS	Cntrl	2.9 uM PFOS	5.8 uM PFOS	11.6 uM PFOS	265 uM PFOS	Cntrl		

**1 dpt**												
	1	2	3	4	5	6	7	8	9	10	11	12
A	1	1	1	0	1	1	1	1	0	1		
B	1	1	1	0	1	1	1	1	0	1		
C	1	1	1	0	1	1	1	1	0	1		
D	1	1	1	0	1	1	1	1	0	1		
E	1	1	1	0	1	1	1	1	0	1		
F	1	1	1	0	1	1	1	1	0	1		
G	1	1	1	0	1	1	1	1	0	1		
H	1	1	1	0	1	1	1	1	0	1		
Avg												

**3 dpt**												
	1	2	3	4	5	6	7	8	9	10	11	12
A	1	1	0	0	1	1	1	0	0	1		
B	1	1	0	0	1	1	1	0	0	1		
C	1	1	0	0	1	1	1	0	0	1		
D	1	1	0	0	1	1	1	0	0	1		
E	1	1	0	0	1	1	1	0	0	1		
F	1	1	0	0	1	1	1	0	0	1		
G	1	1	0	0	1	1	1	0	0	1		
H	1	1	1	0	1	1	1	0	0	1		
Avg												

**5 dpt**												
	1	2	3	4	5	6	7	8	9	10	11	12
A	1	1	0	0	1	1	1	0	0	1		
B	1	0	0	0	1	1	1	0	0	1		
C	1	1	0	0	1	1	1	0	0	1		
D	1	1	0	0	1	1	1	0	0	1		
E	1	1	0	0	1	1	0	0	0	1		
F	1	1	0	0	1	1	1	0	0	1		
G	1	1	0	0	1	1	0	0	0	1		
H	1	1	0	0	1	1	0	0	0	1		

ACHN Analysis

2.9 uM PFOS	d0 Area	d3 Area	Percent change
**A2**	278123.68	266108.843	-0.04319961896
**B2**	163520.616	156060.855	-0.04561969727
**C2**	110312.051	197320.598	0.7887492455
**D2**	82519.487	82717.534	0.002400002802
**E2**	161870.226	167019.442	0.03181076673
**F2**	113612.83	76181.991	-0.329459613
**G2**	72947.227	106153.069	0.4552036228
**H2**	229470.191		
**A8**	215870.979		
**B8**	96382.761		
**C8**	178374.124	125231.574	-0.2979274617
**D8**	270729.934	237392.061	-0.1231406978
**E8**	171970.612	403421.27	
**F8**	175667.485	134143.679	-0.2363773011
**G8**	139887.035	83443.706	-0.4034922107
**H8**	120808.53	116319.469	-0.03715847714
Average	161379.2355	165501.0839	-0.01985095333
Stdev	62750.06426	92695.97334	0.3387277025

**Summary Table**	Average	Stdev
2.9 uM PFOS	-0.01985095333	0.3387277025
5.8 uM PFOS	0.4014588317	0.3705694833
11.6 uM PFOS	0.4928490581	0.5097320953
Cntrl	0.05064488854	0.3271007945

5.8 uM PFOS	d0 Area	d3 Area	Percent change
**A4**	172894.83	221020.195	0.2783505152
**B4**	155796.792		
**C4**	35714.434	84169.877	1.356746771
**D4**	88658.937	87074.563	-0.01787043758
**E4**	51954.269		
**F4**	197122.552		
**G4**	90705.421	133087.429	0.4672488979
**H4**	133153.445		
**A10**	239834.638		
**B10**	126155.792	214880.745	0.7032967063
**C10**	358332.622		
**D10**	89781.202	105360.882	0.173529421
**E10**	120280.405		
**F10**	276275.244	360973.246	0.3065710875
**G10**	201083.487		
**H10**	165303.037	193821.772	0.1725239628
**A8**	169594.051	197056.536	0.1619307095
**B8**	160549.915	232506.908	0.4481907885
**C8**	179232.327		
**D8**	158569.447	216531.135	0.3655287264
Average	158549.6424	186043.9353	0.4014588317
Stdev	75503.95642	80878.06899	0.3705694833

11.6 uM PFOS	d0 Area	d3 Area	Percent change
**A4**	182599.122	185701.854	0.01699204227
**B4**	125825.714	284659.224	1.262329495
**C4**	169594.051	314366.239	0.8536395419
**D4**	128070.244	168471.785	0.3154639184
**E4**	132559.305	118563.999	-0.105577696
**F4**	188408.494		
**G4**	116385.485	239636.591	1.058990354
**H4**	180486.623	209071.373	0.1583760033
**A10**	139160.864	281094.382	1.019924093
**B10**	295815.858	248878.774	-0.1586699385
**C10**	195010.053		
**D10**	120148.374	240626.825	1.002747245
**E10**	253895.959		
**F10**	235741.672	301427.184	0.2786334357
**G10**	332982.636	403355.254	0.2113402033
**H10**	234685.422		
Average	189460.6173	249654.457	0.4928490581
Stdev	65591.41259	75317.80038	0.5097320953

Cntrl	d0 Area	d3 Area	Percent change
**A6**	180420.607		
**B6**	125561.652	89055.031	-0.2907465808
**C6**	183391.309	225443.24	0.2293016568
**D6**	183061.231	66279.652	-0.6379372539
**E6**	176657.719	232572.923	0.3165171854
**F6**	81529.254	83245.659	0.02105262732
**G6**	84631.986	62648.795	-0.2597503856
**H6**	102126.118		
**A12**	196396.38	199565.128	0.01613445217
**B12**	96184.715	142461.643	0.4811255926
**C12**	189794.821	209797.545	0.1053913057
**D12**	199103.019	168999.91	-0.1511936341
**E12**	145696.407	136058.131	-0.06615314817
**F12**	71032.775	58753.875	-0.1728624568
**G12**	207619.03	206562.781	-0.00508743827
**H12**	205242.469		
**A6**	169396.004	240692.841	0.4208885411
**B6**	195604.193	204648.329	0.04623692295
**C6**	285913.52	194019.819	-0.3214038322
**D6**	169065.926	219039.727	0.2955876573
**E6**	157117.104	272116.262	0.7319327754
**F6**	206232.703	183391.309	-0.1107554411
**G6**	178110.062	300436.95	0.6868050386
**H6**	256470.567	234421.36	-0.08597168579
**A12**	250463.148	170320.222	-0.3199789136
**B12**	241551.044		
**C12**	130512.821	172894.83	0.3247344489
**D12**	208939.342		
**E12**	188672.556		
**F12**	73145.274	91893.701	0.2563176809
**G12**	196990.52	205836.609	0.04490616604
**H12**	96118.699	73211.289	-0.2383241787
Average	169773.5305	170937.2139	0.05064488854
Stdev	55742.65907	69027.86805	0.3271007945

Caki-1 Analysis

2.9 uM PFOS	d0 Area	d3 Area	Percent change
**A8**	91761.67	61724.577	-0.3273381249
**B8**	93412.06	54924.971	-0.4120141339
**C8**	109321.817	40401.541	-0.6304347832
**D8**	127806.182	29971.078	-0.7654958662
**E8**	63506.998	68854.26	0.0841995712
**F8**	186758.104	225047.146	0.2050194405
**G8**	199367.082	122524.935	-0.3854304644
**H8**	225113.162	150317.498	-0.3322580667
**A2**	96382.761	65223.403	-0.3232876676
**B2**	77700.349	40731.619	-0.4757858938
**C2**	125825.714	57103.485	-0.5461699903
**D2**	251585.413	101267.915	-0.5974809756
**E2**	92157.764	61328.483	-0.3345272244
**F2**	134671.803	87800.735	-0.3480392106
**G2**	202799.892	117309.703	-0.4215494799
**H2**	76314.022	30301.156	-0.6029411738
**A8**	174743.267	72617.149	-0.5844352103
**B8**	138368.676	47927.318	-0.6536259551
**C8**	142131.565	42514.04	-0.7008824887
**D8**	99353.463	24491.784	-0.7534883711
**E8**	190124.899	122524.935	-0.3555555551
**F8**	119686.265	58489.813	-0.5113072248
**G8**	84896.049	80473.004	-0.05209953881
**H8**	239570.576	168207.723	-0.2978782044
Average	139306.648	80503.26129	-0.421783608
Stdev	55902.53104	49155.13357	0.2437305752

**Summary Table**	Average	Stdev
2.9 uM PFOS	-0.421783608	0.2437305752
5.8 uM PFOS	-0.4209069681	0.176879547
11.6 uM PFOS	-0.1365362297	0.4235901385
Cntrl	-0.4942327624	0.2441848486

5.8 uM PFOS	d0 Area	d3 Area	Percent change
**A4**	125363.605	58885.906	-0.530279095
**B4**	178572.171	141405.394	-0.2081330859
**C4**	128862.432	51690.207	-0.5988729516
**D4**	98957.369	47201.147	-0.5230153401
**E4**	167415.536	95392.527	-0.4302050498
**F4**	198706.926	151373.748	-0.2382059798
**G4**	206034.656		
**H4**	181806.935	125297.59	-0.3108206241
**A10**	81463.238	24557.799	-0.6985413347
**B10**	88196.828	45154.664	-0.4880239457
**C10**	33469.904	17428.116	-0.4792899316
**D10**	122855.013	39081.229	-0.6818914585
**E10**	197122.552	110180.02	-0.4410582712
**F10**	787565.988	507989.964	-0.3549874274
**G10**	172168.659	135662.037	-0.2120398812
**H10**	144046.017	127013.995	-0.1182401454
Average	182037.9893	111887.6229	-0.4209069681
Stdev	168710.9223	118772.5272	0.176879547

11.6 uM PFOS	d0 Area	d3 Area	Percent change
**A3**	122128.841	68788.245	-0.4367567526
**B3**	85424.173		
**C3**	100079.634		
**D3**	57367.548	56971.454	-0.00690449590
**E3**	88724.953	119818.296	0.3504464297
**F3**	124571.418	70636.681	-0.432962375
**G3**	164114.757		
**H3**	200093.253	159097.572	-0.2048828753
**A9**	113612.83		
**B9**	143319.846	54660.908	-0.6186089399
**C9**	88724.953		
**D9**	126947.979	61988.639	-0.5117004659
**E9**	120544.467	119092.124	-0.01204819297
**F9**	171376.471		
**G9**	89715.187	147544.843	0.6445916008
**H9**	196528.411		
Average	124579.6701	95399.86244	-0.1365362297
Stdev	41286.6226	41091.64838	0.4235901385

Cntrl	d0 Area	d3 Area	Percent change
**A6**	170980.378	82915.581	-0.515057915
**B6**	72353.087	3234.764	-0.9552919698
**C6**	103050.336	73475.352	-0.2869955126
**D6**	89187.062	67005.824	-0.2487046608
**E6**	95656.59	26076.158	-0.7273982064
**F6**	133285.476	107473.38	-0.1936602305
**G6**	197452.629	185635.839	-0.0598462024
**H6**	246964.322	159559.681	-0.3539160648
**A12**	134671.803	57103.485	-0.5759803929
**B12**	71494.884	56509.345	-0.2096029556
**C12**	78030.427	2310.546	-0.9703891663
**D12**	142659.69	38619.12	-0.7292919955
**E12**	147478.828	77106.209	-0.4771709943
**F12**	121534.701	79284.723	-0.3476371576
**G12**	276407.275	265646.734	-0.03893002093
**H12**	171442.487	77436.287	-0.5483249902
**A6**	70834.728	33007.795	-0.5340167749
**B6**	126155.792	20728.895	-0.8356881228
**C6**	76181.991	33601.935	-0.5589254815
**D6**	150251.483	52812.472	-0.6485061515
**E6**	172894.83	87668.703	-0.4929362376
**F6**	277529.54	113810.877	-0.5899143673
**G6**	119422.202	62780.826	-0.4742951901
**H6**	225773.318	124571.418	-0.448245616
**A12**	134539.772	52548.41	-0.6094209971
**B12**	90375.343	54792.94	-0.393718041
**C12**	84103.862	25482.018	-0.6970172666
**D12**	109717.91	29839.047	-0.7280385035
**E12**	196396.38	73871.445	-0.6238655468
**F12**	74663.632	72683.165	-0.02652518967
**G12**	179628.42	103842.523	-0.421903711
**H12**	173158.892		
Average	141071.1897	74239.85474	-0.4942327624
Stdev	59118.39246	54520.64141	0.2441848486
