# Supplementary material for: Assessing Perfluorooctane Sulfonate (PFOS) Toxicity and Carcinogenicity Through Zebrafish (Danio rerio) Xenograft Assays
Source: Toxics. 2025 Dec 14;13(12):1077. doi: 10.3390/toxics13121077 (PMC12737301; doi:10.3390/toxics13121077)
Supplement: Supplementary file 1 [file toxics-13-01077-s001.zip › Supplemental Figure S1_Caki-1 Xenografts.pdf]

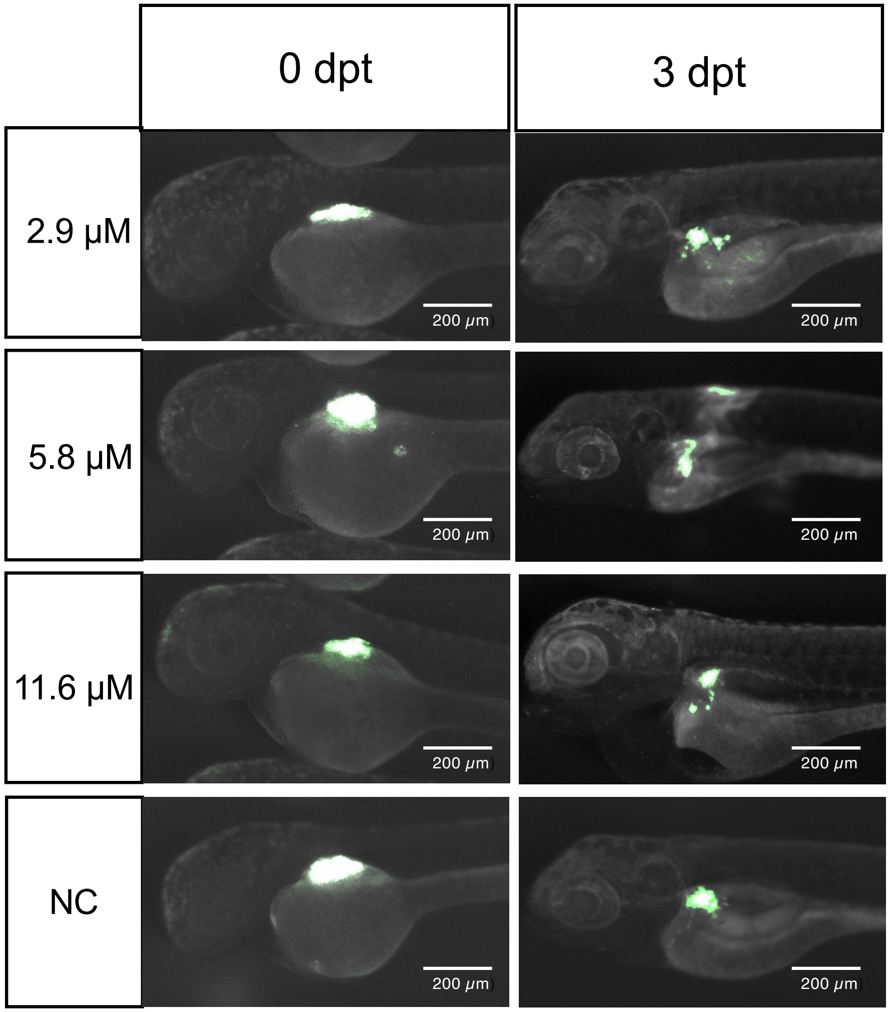

Supplemental Figure S1. Green fluorescent kidney cancer Caki-1 cell xenografts overlaid on mpeg1:mCherry transgenic embryos. GFP labeled cells were injected dorsal to the yolk in the area of the developing kidney of 2dpf embryos. Images at 0 and 3 days post PFOS treatment.
