# Supplementary material for: Assessing Perfluorooctane Sulfonate (PFOS) Toxicity and Carcinogenicity Through Zebrafish (Danio rerio) Xenograft Assays
Source: Toxics. 2025 Dec 14;13(12):1077. doi: 10.3390/toxics13121077 (PMC12737301; doi:10.3390/toxics13121077)
Supplement: Supplementary file 1 [file toxics-13-01077-s001.zip › Supplemental Table S1_PFOS Literature Summary.pdf]

Supplemental Table S1. Summary of literature values for PFOS LC50 and PFOS experiments with zebrafish.

Publication	Analysis Type	PFOS Concentration Range Used	Treatment/Assesment Period	Metric of Interest	PFOS Concentration Endpoint Determined (If Applicable)
Huang et al., 2010	Toxicity	0.5 - 16 uM	6-120 hpf	Survival	LC50 = 4.39 μM (120 hpf)
Ding et al., 2013	Toxicity	Not Specified	0-96 hpf	Survival & LC50	LC50= 102uM (@72hpf), 101uM (@96hpf)
Hagenaars et al., 2011	Developmental	2-200 uM	8 hpf-120hpf	Development malformations	LC50 = 117 uM (@96 hpf)
Jantzen et al., 2016	Behavioral	0-2uM	3-120hpf	Morphometric, Behavior, & Gen	LC50 = 25uM
Martinez et al., 2019	Developmental	0.2-200 uM	Start: 2dpf; End: 5 dpf	Morphological and transcriptor	LC50 = 18.2 μM (120 hpf)
Zheng et al., 2012	Developmental	0-400 uM	0-72 hpf	Survival and developmental to›	LC50 = 137.98 μM (24 hpf), 136 μM (48 hpf), 136 μM (72 hpf)
Yang et al., 2023	Developmental	0-160.28 μM	1.5-96hpf	Survival & toxicity	LC50 = 125.0 μM (72hpf) , 115.15 μM (96 hour)
Ding et al., 2011	Developmental	0-400 uM	0-120 hpf	Survival and morphological cha	LC50 = 158.1 μM (96 hpf), 140.3 uM (120 hpf)
**Limbu et al., 2024**	Toxicity	0-1000 uM	48 hpf - 72hpf	Survival	LC50 = 53 μM (72 hpf)
Tu et al., 2019	Developmental	0.05-5 uM	Start: 3hpf; End: 4 dpf	Bioconcentration and metaboli	LC50 = ~50uM (96 hpf, unpublished result)
Tal and Vogs, 2021	Literature Review	Various	Various	Various	Various
Bland et al., 2017	Biomarker	1-50uM	24-96 hpf	Gene Expression	N/A
Chen et al., 2013	Behavioral	0.5 μM	1-120dpf	Behavioral Deficits	0.5uM
Chen et al., 2014	Developmental	8-32 uM	Start: 0 hpf; End: 96 hpf	Morphological Changes & Gen	16uM
Chen et al., 2016	Hormonal	0-0.5 uM	Start: 8 hpf; End: 5 dpf	Hormone Levels, Gene Express	N/A
Dong et al., 2021	Biomarker & Develo	1uM	4-120hpf	Gene expression & Organ Deve	N/A
Du et al., 2017	Developmental	2-32uM	N/A	Oxidative stress & Apoptosis	LC50 = 7uM (@96hpf)
Fey et al., 2022	Developmental	0.02-15uM	0-5dpf	Mixture Additivity Effects on To	BMD = 4.54 uM
Gaballah et al., 2020	Developmental	0-80uM	0-5dpf	Neurotoxicity	EC50 = 7.5 uM
Gutsfeld et al., 2024	Developmental	0.43-7.86 uM	1-4dpf	Gene Expression	N/A
Haimbaugh et al., 2022	behavioral	0-0.005 uM	<4hpf-5dpf	Gene Expression	N/A (No mortality seen below 20 uM)
Han et al., 2021	Developmental	0.5-5uM	0-5dpf	Metabolic Effects	N/A
Hawkey et al., 2023	Behavioral	0.01-0.1uM	5-120hpf	Neurotoxicity	N/A
Khezri et al., 2017	Behavioral	0-10.92 uM	6-96hpf	gene expression and swim beh	N/A
Menger et al., 2020	Behavioral	0-400 uM	0-144hpf	changes in locomoter activity	EC50 = 3 uM
Min et al., 2024	Behavioral	0-20 uM	4-120hpf	Metabolic and Lipidomic Dysre	N/A
Phelps et al., 2023	Behavioral	Not specified	6-96hpf	Neutrophil Respiratory Burst	AC50 = 2.51 uM
Rericha et al., 2024	Developmental	0.015-100uM	6-120hpf	Transciptomic Changes	BMD80 = 13.8 uM
Truong et al., 2022	Developmental	0.015-100uM	6-120hpf	Larval Photomoter Response &	N/A
Ulhaq et al., 2013a	Developmental	0.06-20uM	0-144hpf	Embryonic Toxicity	EC50 = 3 uM(@144hpf), LC50 = >10uM (@144hpf)
Ulhaq et al., 2013b	Behavioral	0.06-20uM	0-144hpf	Locomotor Behavior	EC50 = 3 uM(@144hpf)
Shi et al., 2008	Developmental	0-10 uM	Start: 4-5hpf; End: 132 hpf	developmental toxicity and ger	N/A

Publication	Analysis Type	PFOS Concentration Range Used	Treatment/Assesment Period	Metric of Interest	PFOS Concentration Endpoint Determined (If Applicable)
Cheng et al, 2016	Developmental	0.5 uM	Start: 8 hpf; End: 5 months pf	lipid metabolism disruption	N/A
Du et al., 2009	Developmental	0-0.5 uM	Start: 14 dpf; End: 84 dpf	fish reproduction via endocrine	No mortality observed
Jantzen et al., 2016	Behavioral	0-2.0 uM	Start: 3 hpf; End: 120 hpf	long term effects of gene expre	N/A
Shi and Zhou, 2010	Developmental	0.4 - 2.0 uM	Start: 4 hpf; End: 96 hpf	Gene expression changes due	N/A
Shi et al., 2009	Developmental	0-0.8 uM	Start: 2hpf; End: 15 dpf	developmental toxicity and ger	No significant effect on survival
Keiter et al., 2012	Developmental	0.0012-0.6 uM	Start: 2-4 hpf; End: 48 hpf	Long term effects on developm	no significant mortality observed in F1, long-term mortality effects seer
Cui et al., 2015	Developmental	0-200 uM	48 hour treatment	Effects on apoptosis and cell c	IC50 = 55.8 uM, IC80 = 113.5 uM
Chen et al., 2018	Developmental	0.01-0.50 uM	Start: 8hpf; End: 120 dpf	Disruption of thryoid structure	N/A
Huang et al., 2021	Developmental	0-40uM	96 hour exposure	Cilia disruption of OBS (a comp	LC50 of OBS = 40.8 uM for adult zebrafish (comparable to PFOS)
Wang et al. 2011	Developmental	0.01-0.50 uM	Start: 8 hpf; End: 5dpf	Sex ratio and maternal related	No mortality observed
Jantzen et al., 2016	Developemental	0.00-2.0 uM	Start: 3hpf; End: 120 hpf	embryonic development and ge	no significant mortality observed
Kalyn et al., 2023	Behavioral	0-2 uM	Start: 72 hpf; End: 120 hpf	Effects on locomotor behaviora	N/A
Zou et al., 2021	Toxicity	0-0.02 uM	48 hours of exposure	comparison if toxicity between	96h LC50 = 34 uM (adult zebrafish) --> reported from another paper
Huang et al., 2022	Toxicity	0.002-0.2 nM	28 day exposure	Comparison between PFOS bic	no fish died
Wang et al., 2022	Behavioral	0.004-1.0 uM	Start: 2hpf; End: 96 hpf	Locomotor behavior changes c	Death rates did not differ between concentrations
Pandelides et al., 2024	Literature Review	Various	Various	Various	Various
Liu et al., 2022	Toxicity	0.002-0.02 uM	Start: 4-6 hpf; End: 120 hpf	cardiotoxicity in early developn	N/A
Sant et al., 2017	Developmental	0-64 uM	Start: 3hpf; End: 168 hpf	Disruption of pancreatic organ	No mortality observed
Gust et al., 2025	Chronic Exposure	0-0.2uM	0-180 dpf	Bioconcentration factors	N/A
Menger et al., 2020	Behavioral	0-400uM	0-144hpf	Swimming behaviour, behaviou	N/A
Sant et al., 2021	Developmental	0, 16, 32uM	1-30dpf	Pancreatic islet morphometry,	N/A
Shankar et al., 2025	Toxicology	0-100uM	6-144hpf	Transcriptomics-based point o	N/A
Satbhai et al., 2025	Developmental	16uM	3-72hpf	Gene expression	N/A
Mylroie et al., 2021	Toxicology	0 - 7.92 μM	0-120hpf	Survival, spinal curvature, swin	LC50 = 4.50 μM (120hpf, chorinated), and 3.68 μM (120hpf, dechorinat
Hagenaars et al., 2014	Behavioral	0-200 uM	Start: 60 min pf; End: 144 hpf	Changes in swim bladder and s	LC50 = 12.5 uM (144 hpf)
Ma et al., 2024	Developmental	0.002-2 uM	Start: 2 hpf; End: 72 hpf	Oxidative stress mediated card	N/A
Zoodsma et al., 2024	Behavioral	2-20 uM	0-5dpf	Impact on ability to capture pre	N/A
Lee et al., 2022	Behavioral	0-20 uM	Start: 4 hpf; End: 120 hpf	Seizurogenic effect on larvae	No significant mortality
Shi et al., 2009	Developmental	0-1 uM	Start: 4-5 hpf; End: 192 hpf	Altered protein expression due	no significant mortality
Du et al., 2008	Developmental	0.02-0.50 uM	14-70 dpf	Growth, development, and hepa	No significant effect on survival
Wang et al., 2025	Developmental	0.3mg/L & 30mg/L	0-21dpf	Survival and morphological cha	N/A

Publication	Analysis Type	PFOS Concentration Range Used	Treatment/Assesment Period	Metric of Interest	PFOS Concentration Endpoint Determined (If Applicable)
Christou et al., 2021	Developmental	0.55uM & 3.83uM	exposure: 6-96hpf, assessment: 0-1[illegible]	Survival, growth measurement[illegible]	N/A
Gust et al., 2024	Chronic Exposure	0-100ug/L	0-180 dpf	Survival, growth measurement[illegible]	N/A
