# Supplementary material for: Assessing Perfluorooctane Sulfonate (PFOS) Toxicity and Carcinogenicity Through Zebrafish (Danio rerio) Xenograft Assays
Source: Toxics. 2025 Dec 14;13(12):1077. doi: 10.3390/toxics13121077 (PMC12737301; doi:10.3390/toxics13121077)
Supplement: Supplementary file 1 [file toxics-13-01077-s001.zip › Supplemental Table S2, S3, S4, and S5_Statistics.pdf]

Supplemental Table S2. ACHN ordinary one-way ANOVA statistics with uncorrected Fisher's LSD tests for multiple comparisons.

**Distribution assumption**	Normal (Gaussian)

**ANOVA summary**	
F	6.107
P value	0.0011
P value summary	**
Significant diff. among means (P < 0.05)?	Yes
R squared	0.2432

**ANOVA table**	**SS**	**DF**	**MS**	**F (DFn, DFd)**	**P value**
Treatment (betw	2.625	3	0.8751	F (3, 57) = 6.107	P=0.0011
Residual (within	8.168	57	0.1433		
Total	10.79	60			

Homogeneity of Variances

**Brown-Forsythe test**		**Bartlett's test**	
F (DFn, DFd)	1.735 (3, 57)	Bartlett's statistic (corrected)	3.512
P value	0.17	P value	0.3192
P value summary	ns	P value summary	ns
Are SDs significantly different (P < 0.05)?	No	Are SDs significantly different (P < 0.05)?	No

Normality of Residuals

Test name	Statistics	P value	Passed normality test (alpha=0.05)?	P value summary
**D'Agostino-Pearson omnibus (K2)**	4.041	0.1326	Yes	ns
Anderson-Darling (A2*)	0.9772	0.0131	No	*
**Shapiro-Wilk (W)**	0.959	0.0395	No	*
Kolmogorov-Smirnov (distance)	0.1342	0.0081	No	**

Multiple Comparisons

Number of families	1
Number of comparisons per family	6
Alpha	0.05

**Uncorrected Fisher's LSD**	**Mean diff.**	**95.00% CI of diff.**	**elow threshold?**	**Summary**	**Individual P Value**	
2.9 uM PFOS vs. 5.8 uM PFOS	-0.4213	-0.7377 to -0.1049	Yes	**	0.01	A-B
2.9 uM PFOS vs. 11.6 uM PFOS	-0.5127	-0.8222 to -0.2032	Yes	**	0.0016	A-C
2.9 uM PFOS vs. Control	-0.0705	-0.3350 to 0.1941	No	ns	0.5957	A-D
5.8 uM PFOS vs. 11.6 uM PFOS	-0.09139	-0.4078 to 0.2250	No	ns	0.5653	B-C
5.8 uM PFOS vs. Control	0.3508	0.07816 to 0.6235	Yes	*	0.0126	B-D
11.6 uM PFOS vs. Control	0.4422	0.1777 to 0.7068	Yes	**	0.0015	C-D

**Test details**	**Mean 1**	**Mean 2**	**Mean diff.**	**SE of diff.**	**n1**	**n2**	**t**	**DF**
2.9 uM PFOS vs. 5.8 uM PFOS	-0.01985	0.4015	-0.4213	0.158	12	11	2.666	57
2.9 uM PFOS vs. 11.6 uM PFOS	-0.01985	0.4928	-0.5127	0.1545	12	12	3.317	57
2.9 uM PFOS vs. Control	-0.01985	0.05064	-0.0705	0.1321	12	26	0.5336	57
5.8 uM PFOS vs. 11.6 uM PFOS	0.4015	0.4928	-0.09139	0.158	11	12	0.5784	57
5.8 uM PFOS vs. Control	0.4015	0.05064	0.3508	0.1362	11	26	2.577	57
11.6 uM PFOS vs. Control	0.4928	0.05064	0.4422	0.1321	12	26	3.347	57

**Compact letter display**	
11.6 uM PFOS	A
5.8 uM PFOS	A
Control	B
2.9 uM PFOS	B

Data summary

Number of treatments (columns)	4
Number of values (total)	61

Supplemental Table S3. Caki-1 Brown-Forsythe ANOVA statistics with unpaired Welch's t-tests for multiple comparisons.

**Distribution assumption**	Normal (Gaussian)
**Brown-Forsythe ANOVA test**	
F* (DFn, DFd)	3.412 (3.000, 20.64)
P value	0.0367
P value summary	*
Significant diff. among means (P < 0.05)?	Yes
**Welch's ANOVA test**	
W (DFn, DFd)	2.048 (3.000, 27.02)
P value	0.1307
P value summary	ns
Significant diff. among means (P < 0.05)?	No
Multiple Comparisons	
Number of families	1
Number of comparisons per family	6
Alpha	0.05

**Unpaired t with Welch's correction**	**Mean diff.**	**95.00% CI of diff.**	**Below threshold?**	**Summary**	**Individual P Value**	
2.9 uM PFOS vs. 5.8 uM PFOS	-0.0008766	-0.1378 to 0.1361	No	ns	0.9897	A-B
2.9 uM PFOS vs. 11.6 uM PFOS	-0.2852	-0.6186 to 0.04807	No	ns	0.0857	A-C
2.9 uM PFOS vs. Control	0.07245	-0.06079 to 0.2057	No	ns	0.2799	A-D
5.8 uM PFOS vs. 11.6 uM PFOS	-0.2844	-0.6164 to 0.04767	No	ns	0.0852	B-C
5.8 uM PFOS vs. Control	0.07333	-0.05497 to 0.2016	No	ns	0.2543	B-D
11.6 uM PFOS vs. Control	0.3577	0.02637 to 0.6890	Yes	*	0.0371	C-D

**Test details**	**Mean 1**	**Mean 2**	**Mean diff.**	**SE of diff.**	**n1**	**n2**	**t**	**DF**
2.9 uM PFOS vs. 5.8 uM PFOS	-0.4218	-0.4209	-0.0008766	0.06753	24	15	0.01298	36.05
2.9 uM PFOS vs. 11.6 uM PFOS	-0.4218	-0.1365	-0.2852	0.1497	24	9	1.905	10.06
2.9 uM PFOS vs. Control	-0.4218	-0.4942	0.07245	0.06632	24	31	1.092	49.65
5.8 uM PFOS vs. 11.6 uM PFOS	-0.4209	-0.1365	-0.2844	0.1484	15	9	1.916	9.701
5.8 uM PFOS vs. Control	-0.4209	-0.4942	0.07333	0.06332	15	31	1.158	37.03
11.6 uM PFOS vs. Control	-0.1365	-0.4942	0.3577	0.1479	9	31	2.419	9.594

**Compact letter display**	
11.6 uM PFOS	A
5.8 uM PFOS	A B
2.9 uM PFOS	A B
Control	B

**Data summary**	
Number of treatments (columns)	4
Number of values (total)	79

Supplemental Table S4. Caki-1 ordinary one-way ANOVA statistics with multiple Fisher's LSD comparison tests and a violated homogeneity of variance assumption. This statistical data was not used. See Supplemental Table S3 for used Caki-1 statistics.

**Distribution assumption**	Normal (Gaussian)

**ANOVA summary**	
F	4.443
P value	0.0063
P value summary	**
Significant diff. among means (P < 0.05)?	Yes
R squared	0.1509

**ANOVA table**	**SS**	**DF**	**MS**	**F (DFn, DFd)**	**P value**
Treatment (between columns)	0.8936	3	0.2979	F (3, 75) = 4.443	P=0.0063
Residual (within columns)	5.029	75	0.06705		
Total	5.922	78			

Homogeneity of Variances

**Brown-Forsythe test**		**Bartlett's test**	
F (DFn, DFd)	2.800 (3, 75)	Bartlett's statistic (corrected)	8.843
P value	0.0457	P value	0.0315
P value summary	*	P value summary	*
Are SDs significantly different (P < 0.05)?	Yes	Are SDs significantly different (P < 0.05)?	Yes

Normality of Residuals

Test name	Statistics	P value	Passed normality test (alpha=0.05)?	P value summary
**D'Agostino-Pearson omnibus (K2)**	5.734	0.0569	Yes	ns
Anderson-Darling (A2*)	0.5062	0.1959	Yes	ns
**Shapiro-Wilk (W)**	0.9747	0.1174	Yes	ns
Kolmogorov-Smirnov (distance)	0.07932	0.1	Yes	ns

Multiple Comparisons

Number of families	1
Number of comparisons per family	6
Alpha	0.05

**Uncorrected Fisher's LSD**	**Mean diff.**	**95.00% CI of diff.**	**Below threshold?**	**Summary**	**Individual P Value**	
2.9 uM PFOS vs. 5.8 uM PFOS	-0.0008766	-0.1707 to 0.1689	No	ns	0.9918	A-B
2.9 uM PFOS vs. 11.6 uM PFOS	-0.2852	-0.4869 to -0.08363	Yes	**	0.0062	A-C
2.9 uM PFOS vs. Control	0.07245	-0.06780 to 0.2127	No	ns	0.3068	A-D
5.8 uM PFOS vs. 11.6 uM PFOS	-0.2844	-0.5019 to -0.06688	Yes	*	0.0111	B-C
5.8 uM PFOS vs. Control	0.07333	-0.08891 to 0.2356	No	ns	0.3708	B-D
11.6 uM PFOS vs. Control	0.3577	0.1624 to 0.5530	Yes	***	0.0005	C-D

**Test details**	**Mean 1**	**Mean 2**	**Mean diff.**	**SE of diff.**	**n1**	**n2**	**t**	**DF**
2.9 uM PFOS vs. 5.8 uM PFOS	-0.4218	-0.4209	-0.0008766	0.08523	24	15	0.01029	75
2.9 uM PFOS vs. 11.6 uM PFOS	-0.4218	-0.1365	-0.2852	0.1012	24	9	2.818	75
2.9 uM PFOS vs. Control	-0.4218	-0.4942	0.07245	0.0704	24	31	1.029	75
5.8 uM PFOS vs. 11.6 uM PFOS	-0.4209	-0.1365	-0.2844	0.1092	15	9	2.605	75
5.8 uM PFOS vs. Control	-0.4209	-0.4942	0.07333	0.08144	15	31	0.9004	75
11.6 uM PFOS vs. Control	-0.1365	-0.4942	0.3577	0.09804	9	31	3.648	75

Compact letter display

11.6 uM PFOS	A
5.8 uM PFOS	A B
2.9 uM PFOS	B
Control	B

Data summary

Number of treatments (columns)	4
Number of values (total)	79

Supplemental Table S5. Post-hoc Dunnett's tests for ACHN and Caki-1 data sets from respective ANOVA testing methods referenced in the manuscript (α = 0.05).

**ACHN**								
Number of families	1							
Number of comparisons per family	3							
Alpha	0.05							
**Dunnett's multiple comparisons test**	**Mean diff.**	**95.00% CI of diff.**	**Below threshold?**	**Summary**	**Adjusted P Value**	**D-?**		
Control vs. 2.9 uM PFOS	0.0705	-0.2521 to 0.3931	No	ns	0.9249	A	2.9 uM PFOS	
Control vs. 5.8 uM PFOS	-0.3508	-0.6833 to -0.01836	Yes	*	0.0358	B	5.8 uM PFOS	
Control vs. 11.6 uM PFOS	-0.4422	-0.7648 to -0.1196	Yes	**	0.0043	C	11.6 uM PFOS	
**Test details**	**Mean 1**	**Mean 2**	**Mean diff.**	**SE of diff.**	**n1**	**n2**	**q**	**DF**
Control vs. 2.9 uM PFOS	0.05064	-0.01985	0.0705	0.1321	26	12	0.5336	57
Control vs. 5.8 uM PFOS	0.05064	0.4015	-0.3508	0.1362	26	11	2.577	57
Control vs. 11.6 uM PFOS	0.05064	0.4928	-0.4422	0.1321	26	12	3.347	57
**Caki-1**								
Number of families	1							
Number of comparisons per family	3							
Alpha	0.05							
**Dunnett's T3 multiple comparisons test**	**Mean diff.**	**95.00% CI of diff.**	**Below threshold?**	**Summary**	**Adjusted P Value**	**D-?**		
Control vs. 2.9 uM PFOS	-0.07245	-0.2361 to 0.09117	No	ns	0.6216	A	2.9 uM PFOS	
Control vs. 5.8 uM PFOS	-0.07333	-0.2314 to 0.08472	No	ns	0.5785	B	5.8 uM PFOS	
Control vs. 11.6 uM PFOS	-0.3577	-0.7759 to 0.06055	No	ns	0.0985	C	11.6 uM PFOS	
**Test details**	**Mean 1**	**Mean 2**	**Mean diff.**	**SE of diff.**	**n1**	**n2**	**t**	**DF**
Control vs. 2.9 uM PFOS	-0.4942	-0.4218	-0.07245	0.06632	31	24	1.092	49.65
Control vs. 5.8 uM PFOS	-0.4942	-0.4209	-0.07333	0.06332	31	15	1.158	37.03
Control vs. 11.6 uM PFOS	-0.4942	-0.1365	-0.3577	0.1479	31	9	2.419	9.594
